# Which Imaging Modality Is Superior for Prediction of Response to Neoadjuvant Chemotherapy in Patients with Triple Negative Breast Cancer?

**DOI:** 10.1155/2013/964863

**Published:** 2013-02-11

**Authors:** Jordan J. Atkins, Catherine M. Appleton, Carla S. Fisher, Feng Gao, Julie A. Margenthaler

**Affiliations:** ^1^Department of Surgery, Washington University School of Medicine, 660 S. Euclid Avenue, Campus Box 8109, St. Louis, MO 63110, USA; ^2^Department of Radiology, Washington University School of Medicine, St. Louis, MO 63110, USA; ^3^Department of Surgery, University of Pennsylvania, Philadelphia, PA 19104, USA; ^4^Division of Biostatistics, Washington University School of Medicine, St. Louis, MO 63110, USA

## Abstract

*Background and Objectives*. Triple negative breast cancer (TNBC) has been shown to be generally chemosensitive. We sought to investigate the utility of mammography (MMG), ultrasonography (US), and breast magnetic resonance imaging (MRI) in predicting residual disease following neoadjuvant chemotherapy for TNBC. *Methods*. We identified 148 patients with 151 Stage I–III TNBC treated with neoadjuvant chemotherapy. Residual tumor size was estimated by MMG, US, and/or MRI prior to surgical intervention and compared to the subsequent pathologic residual tumor size. Data were compared using chi-squared test. *Results*. Of 151 tumors, 44 (29%) did not have imaging performed prior to surgical treatment. Thirty-eight (25%) tumors underwent a pathologic complete response (pCR), while 113 (75%) had residual invasive disease. The imaging modality was accurate to within 1 cm of the final pathologic residual disease in 74 (69%) cases and within 2 cm in 94 (88%) cases. Groups were similar with regards to patient age, race, tumor size and grade, and clinical stage (*P* > 0.05). Accuracy to within 1 cm was the highest for US (83%) and the lowest for MMG (56%) (*P* < 0.05). *Conclusions*. Breast US and MRI were more accurate than MMG in predicting residual tumor size following neoadjuvant chemotherapy in patients with TNBC. None of the imaging modalities were predictive of a pCR.

## 1. Introduction

Breast cancer is the most common cancer in women in the USA, but it is a heterogeneous disease and treatment recommendations vary accordingly. The expressions of steroid hormone receptors such as estrogen receptor (ER) and progesterone receptor (PR), and the oncogene ErbB-2/human epidermal growth factor receptor 2 (HER-2) are important factors in distinguishing breast cancer subtypes. Triple negative breast cancer (TNBC), which is characterized by a lack of ER, PR, and HER-2 expressions, comprises approximately 11%–20% of all newly diagnosed breast cancers [1–5]. Previous studies demonstrate that patients with TNBC have a poorer outcome compared with other subtypes of breast cancer [1, 6–10]. The risk of recurrence for patients with TNBC peaks within the first 3 years following diagnosis and treatment, and the majority of deaths take place within the first 5 years after initial treatment [11–14]. 

Directed therapy options for treating TNBC are limited as these tumors lack a therapeutic target that can be treated with hormone therapy or trastuzumab. As a result, chemotherapy is the standard method used to treat these patients [15–17]. Although randomized studies of neoadjuvant versus adjuvant chemotherapy have failed to demonstrate a survival benefit in either arm, complete pathologic response (pCR) following neoadjuvant chemotherapy has been shown to be a good prognostic marker for patient outcomes [16, 17].

An accurate assessment of the extent of residual disease following neoadjuvant therapy is critical for surgical decision-making. This is generally accomplished with a combination of clinical breast examination with or without some form of breast imaging, most commonly including mammography (MMG), ultrasonography (US), and/or magnetic resonance imaging (MRI). Although these imaging modalities have been shown to be very effective in accurately measuring the tumor size at the time of breast cancer diagnosis, their accuracy in evaluating response to neoadjuvant therapy is less clear [18–21]. We sought to investigate the accuracy of MMG, US, and MRI in predicting the extent of breast residual disease in patients with TNBC undergoing neoadjuvant chemotherapy. We specifically selected this subtype of breast cancer for two primary reasons. First, we were interested in examining a population where pCR was likely to be significantly higher, which would permit a comparative evaluation of various imaging modalities at predicting pCRs. Secondly, compared to other invasive malignancies, such as invasive lobular cancer, TNBC tends to manifest more discrete and quantifiable imaging features; specifically, they tend to manifest as a mass, rather than architectural distortion [22]. 

## 2. Materials and Methods

Institutional review board approval was obtained prior to the commencement of this retrospective study. Written informed consent of patients was not required. The prospectively maintained surgical database at Washington University/Barnes Jewish Hospital was queried from January 1, 2000, to January 31, 2010 to identify all patients with a diagnosis of Stage I–III biopsy-proven invasive TNBC. Patients were divided according to receipt of neoadjuvant chemotherapy, adjuvant chemotherapy, or none/unknown chemotherapy. All patients who were clinically staged as having a Stage I–III TNBC and who received neoadjuvant chemotherapy were included in this study. The precise regimen of neoadjuvant chemotherapy varied and was at the discretion of the treating medical oncologist per the institutional standard of care.

Diagnostic imaging following neoadjuvant chemotherapy was performed to evaluate tumor size after the last cycle of chemotherapy, 1 to 4 weeks prior to surgical intervention. The method of imaging was at the discretion of the treating surgeon. Thus, not all patients were evaluated by imaging, and some patients had a combination of imaging, including MMG, US, and/or MRI. All diagnostic imaging was performed at the Breast Health Center at the Alvin J. Siteman Cancer Center. The Breast Health Center is fully certified according to the federal Mammography Quality Standards Act. Breast imaging examinations were performed using standard techniques by one of our dedicated breast radiologists. For analysis purposes, the longest measured diameter of residual tumor documented by any of the performed imaging modalities was utilized as the final measure of residual tumor prior to surgical intervention. 

ER status, PR status, and HER-2/neu status were determined by core biopsy prior to systemic therapy. ER and PR statuses were determined by standard immunohistochemical methods. Tumors with less than 1% stained cells were considered to have negative receptor status. HER-2/neu status was assessed by immunohistochemistry only if the results were 0 or 1+ staining and by fluorescence in situ hybridization (FISH) confirmation if 2+ immunohistochemistry staining was present. All patients included in the analysis had known HER-2/neu status. Final pathologic assessment of tumor residual volume was performed by 1 of 4 dedicated breast pathologists. The tissue specimen was serially sectioned at 3 to 5 mm intervals in the anteroposterior plane perpendicular to the mediolateral axis and stained by hematoxylin and eosin. Detailed examination was performed, including documentation of the residual invasive or *in situ* tumor size, if present. Patients were considered to have a pCR if no residual invasive tumor was present in the final pathology. For analysis purposes, the longest measured diameter of residual invasive tumor was utilized as the final pathologic tumor size.

 Descriptive statistics were utilized for data summary. Data were compared using Fisher's exact and chi-squared tests. All analyses were performed with SAS version 9 (SAS Institute, Cary, NC). *P* values < 0.05 were considered statistically significant.

## 3. Results

### 3.1. Study Population and Pathologic Outcomes

There were 493 patients with TNBC treated at our institution during the study period. Of these, 148 patients had 151 Stage I–III TNBCs treated with neoadjuvant chemotherapy; all met the inclusion criteria and were considered evaluable.  Table 1 provides the patient and tumor characteristics for the cohort. Thirty-eight (25%) TNBCs experienced a pCR, while 113 (75%) final pathologies revealed residual invasive disease. Of the 38 cases with a pCR, 33 were yT0N0 and 5 were yTisN0. There were no differences between cases with a pCR and those with residual disease on final pathology with respect to patient age, race, pre-therapy clinical tumor size, tumor grade, pretherapy clinical nodal status, or pretherapy clinical stage (*P* > 0.05, Table 2). We were unable to determine the posttherapy clinical tumor size clearly in the retrospective chart review. 

### 3.2. Imaging and Prediction of pCR

Of 151 tumors, 44 (29%) were not imaged prior to surgical treatment, including 16 (36%) in which breast-conserving therapy was performed and 28 (64%) in which mastectomy was performed. The most common reason cited in the medical record for not performing imaging prior to surgical intervention was a planned mastectomy (*N* = 28); the surgeons' notes reflected that the imaging would not alter the planned course. However, the rationale for deferred imaging in the 16 patients undergoing breast-conserving therapy is uncertain and the posttherapy clinical stage was not reliably recorded. 

**Figure 1:**
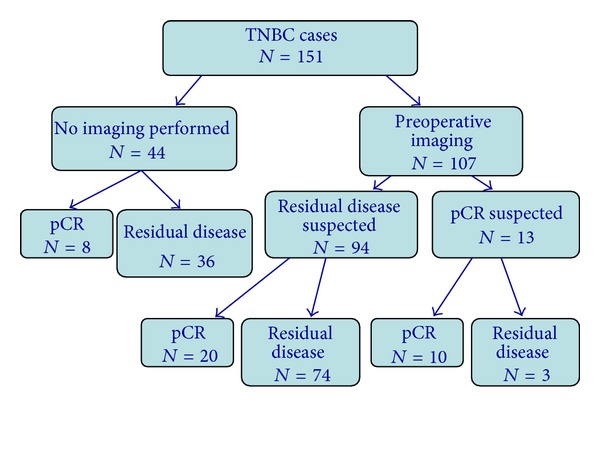
Imaging and pathologic outcomes of 148 patients with 151 Stage I–III triple negative breast cancers (TNBCs) undergoing neoadjuvant chemotherapy prior to definitive surgical intervention.

The remaining 107 (71%) did have one or more imaging studies performed following neoadjuvant chemotherapy but prior to surgical treatment. For these 107 tumors, the method of surgical intervention included 65 (61%) in which breast-conserving therapy was performed and 42 (39%) in which mastectomy was performed. In the 38 cases where a pCR was observed, imaging accurately predicted the pCR for 10 (26%) but residual disease was inaccurately suspected by imaging in 20 (53%); imaging was not performed in 8 (21%). Of 113 cases where residual invasive disease was seen on final pathology, imaging accurately predicted the presence of residual disease in 74 (65%), but inaccurately predicted a pCR for 3 (3%); imaging was not performed in 36 (32%). There were no differences between cases that were not imaged prior to surgical treatment (*N* = 44) and those cases where imaging was utilized prior to surgical treatment (*N* = 107) with respect to patient age, race, pre-therapy clinical tumor size, tumor grade, pre-therapy clinical nodal status, or pre-therapy clinical stage (*P* > 0.05).  Figure 1 illustrates the imaging and pathologic outcomes of the 151 TNBC cases.

### 3.3. Imaging Accuracy

Overall, 107 TNBC cases underwent one or more imaging studies prior to definitive surgical therapy. The imaging modalities utilized included MMG only in 50 (47%), US only in 16 (15%), MRI only in 3 (3%), MMG and US in 32 (30%), MMG and MRI in 2 (2%), US and MRI in 2 (2%), and MMG, US, and MRI in 2 (2%). The imaging modality accurately predicted the burden of final pathologic residual disease to within 1 cm in 74 (69%, 95% CI 62–76%) cases and to within 2 cm in 94 (88%, 95% CI 83–93%) cases. Accuracy to within 1 cm was the highest for US (83%, 95% CI 77–89%) and MRI (78%, 95% CI 71–85%), which were significantly more accurate than MMG (56%, 95% CI 46–64%) (*P* < 0.05). 

Sensitivity and specificity calculations were performed in the 38 patients with a pCR to determine whether a specific imaging modality was superior in predicting this response. Sensitivity and specificity calculations for MRI are limited by the small sample size. No imaging modality was superior with respect to prediction of a pCR (sensitivity and specificity of 36% and 96% for US, 33% and 99% for MRI, and 28% and 87% for MMG; *P* > 0.05). 

## 4. Discussion 

TNBC is an aggressive form of breast cancer with a poorer prognosis compared to other subtypes. However, TNBC is particularly chemosensitive with high pCR rates in the neoadjuvant setting compared to the other breast cancer subtypes [6–10]. An accurate evaluation of residual disease following neoadjuvant chemotherapy is necessary for surgical planning, in order to best select candidates for breast conservation and optimize cosmetic results. In the current study, we evaluated the accuracy of MMG, US, and MRI in estimating residual disease and predicting pCR for a cohort of patients undergoing neoadjuvant chemotherapy for TNBC. We found that US and MRI were superior to MMG in accurately predicting the size of the residual invasive component. We specifically restricted our analysis to this subtype of breast cancer in order to maximize the number of patients likely to experience a pCR and because the imaging findings are typically more easily quantified than other subtypes of breast cancer. Similar to other studies, we did observe a high pCR rate of 25%, though no imaging study was superior in predicting a pCR.

There currently is no clear consensus regarding the best method for accurately assessing residual tumor size following neoadjuvant chemotherapy. Physical examination alone has revealed mixed results. While several studies report that physical examination is accurate in estimating residual tumor size, other studies have shown that it is not reliable [23, 24]. Physical examination is further limited by the inability to distinguish irregular or poorly defined margins or fibrosis/necrosis versus residual tumor. We were unable to document the clinical stage following neoadjuvant chemotherapy in this retrospective study. However, using physical examination alone is limited in the modern era of neoadjuvant chemotherapy administration, whereby many patients have earlier disease and smaller tumors that may not be reliably followed as response occurs. This is particularly true for patients with TNBC who often have the most dramatic responses clinically. 

Thus, a reliable imaging modality is imperative. The use of mammography and ultrasonography for measuring residual tumor size after neoadjuvant chemotherapy has been reported previously [23, 24]. Keune et al. [25] recently compared the accuracy of mammography and ultrasonography in predicting pathologic response after neoadjuvant chemotherapy. Breast ultrasound was more accurate than mammography; ultrasound was able to size the final disease in 91.3% compared to only 51.9% when mammography was used (*P* < 0.001). However, similar to our study, there was no difference in the ability of mammography and ultrasound to predict a pCR. Huber et al. [26] found that the accuracy of mammography in predicting residual tumor disease may depend on the initial mammographic appearance of the tumor. For tumors whose margins could be clearly delineated from the adjacent breast tissue by more than 50% on the baseline mammogram, the diagnostic reliability of posttreatment mammography was high (*r* = 0.77). Ill-defined masses on mammography had a lower correlation with the final pathologic findings (*r* = −0.19) [26]. TNBC is typically welldefined on mammography; however, MMG was the least accurate imaging modality in predicting residual tumor size in our cohort of patients. One hypothesis is that the presence of dead tumor may create a persistent density on MMG accounting for this finding. 

Compared with traditional breast imaging, MRI has been shown to be more accurate in determining the local extent of disease in newly diagnosed breast cancer [27]. Whether this translates to a similarly higher rate of prediction of residual disease following neoadjuvant chemotherapy administration remains unclear. Data suggest that MRI is more accurate compared with palpation and traditional breast imaging in predicting response to neoadjuvant chemotherapy [28, 29]. However, as is the case when MRI is utilized for initial evaluation of extent of disease, concerns over possible overestimation of disease have also been reported [30]. Guarneri et al. [31] directly compared MRI and US in 108 patients undergoing neoadjuvant chemotherapy and found no significant difference in the ability of MRI over US in the prediction of residual disease (*r* = 0.53 and *r* = 0.66, resp.). In contrast, Yeh et al. [32] conducted a prospective study comparing the relative accuracy of palpation, MMG, US, and MRI (19%, 26%, 35%, and 71%, resp.; *P* < 0.002) and found MRI to be superior in predicting the final pathologic disease burden. Further, they did not find any significant tendency for under- or overestimation of disease between the four approaches.

Our study has several limitations. This is a retrospective study, and therefore inherent selection bias is difficult to control. For instance, we cannot determine whether certain neoadjuvant regimens influence response to therapy and subsequent accuracy of the performed imaging. The most common regimens were adriamycin and/or taxane based but the cycle length and specifics of treatment were at the discretion of the treating medical oncologist. Regardless, this is a fair reflection of the chemotherapy practices to date in the setting of TNBC. We also could not control the frequency, consistency, or type of imaging used for each patient. The major limitation of our analysis is the fact that the numbers of MRI utilized overall were very low in our cohort. Further, as we previously noted, a fair number of patients did not have any final imaging prior to surgery, including 16 patients who underwent breast-conserving procedures. It is unclear from the retrospective records how the final surgical planning was conducted; we presume that this was by clinical examination alone, but we were unable to verify post-therapy clinical tumor size retrospectively. Finally, all breast imaging studies are read by dedicated breast radiologists at our institution, likely improving the accuracy of every imaging modality utilized. Thus, our institutional results may not be generalizable to other institutions. 

## 5. Conclusions

In the current study, we found that US and MRI were superior to MMG in accurately predicting residual disease. However, no specific imaging modality was superior in predicting a pCR. Further, the potential increased accuracy of MRI must be counter-balanced with its increased cost compared to traditional breast imaging, as well as the need for intravenous contrast administration. Because the utilization of MRI was very low in our cohort, further recommendations regarding its use must be explored prospectively. In the interim, we propose that breast US represents the most accurate, affordable, noninvasive, and safe modality to assess response to preoperative chemotherapy in patients with TNBC. Prospective studies would be helpful in overcoming the limitations of this and other retrospective analyses and in determining whether the most viable imaging modality varies according to tumor biology. 

## Figures and Tables

**Table 1:** Patient and tumor characteristics of 148 patients with 151 triple negative breast cancers treated with neoadjuvant chemotherapy between 2000 and 2010.

Characteristic	*N* = 148/151* (%)
Age	
<50	93 (62)
≥50	55 (38)
Race	
Caucasian	86 (58)
African-American	57 (39)
Other	5 (3)
Clinical T stage	
T1	22 (15)
T2	73 (48)
T3	47 (31)
Unknown	9 (6)
Nuclear grade	
Grade 1	2 (1)
Grade 2	16 (11)
Grade 3	125 (83)
Unknown	8 (5)
Clinical N status	
N0	62 (41)
N1	63 (41)
N2	10 (7)
N3	9 (6)
Unknown	7 (5)
Clinical stage	
1	10 (7)
2	83 (55)
3	45 (30)
Unknown	13 (8)
Pathologic stage	
0^#^	38 (25)
1	26 (17)
2	50 (33)
3	37 (25)
Unknown	0 (0)
Neoadjuvant regimen	
Taxane-based	129 (87)
Nontaxane^$^	19 (13)
Surgical intervention	
BCT	83 (55)
Mastectomy	68 (45)

BCT: breast conservation therapy.

*N = 148 for patient characteristics (age, race, and neoadjuvant regimen utilized) and *N* = 151 for all tumor characteristics and type of surgical intervention.

^#^Pathologic Stage 0 includes 33 cases with yT0N0 and 5 cases with yTisN0 final pathology.

^$^Nontaxane regimens included cisplatin- and epirubicin-based regimens.

**Table 2:** Association of patient and tumor characteristics comparing residual disease versus no residual disease (pathologic complete response, pCR) following neoadjuvant chemotherapy in 148 patients with 151 triple negative breast cancers.

Characteristic (pretreatment)	Residual disease *n* = 113 (%)	pCR *n* = 38 (%)	*P* value
Age*			
<50	67 (61)	25 (66)	NS
≥50	43 (39)	13 (34)	
Race*			
Caucasian	66 (60)	20 (53)	
African-American	41 (37)	16 (42)	NS
Other	3 (3)	2 (5)	
Clinical T stage			
T1	16 (14)	6 (16)	
T2	51 (45)	22 (58)	NS
T3	37 (33)	10 (26)	
Unknown	9 (8)	0 (0)	
Nuclear grade			
Grade 1	2 (2)	0 (0)	
Grade 2	15 (13)	1 (3)	NS
Grade 3	90 (80)	35 (92)	
Unknown	6 (5)	2 (5)	
Clinical N status			
N0	43 (39)	19 (50)	
N1	48 (42)	15 (39)	
N2	7 (6)	3 (8)	NS
N3	8 (7)	1 (3)	
Unknown	7 (6)	0 (0)	
Clinical Stage			
1	6 (5)	4 (11)	
2	58 (51)	25 (66)	NS
3	38 (34)	7 (18)	
Unknown	11 (10)	2 (5)	
Neoadjuvant regimen^#^			
Taxane-based	96 (85)	36 (95)	NS
Nontaxane^$^	17 (15)	2 (5)	

*N = 148 for patients characteristics (age, race).

^#^ *N* = 151 overall but includes 3 patients with two cancer cases—all 3 received taxane-based regimens.

^$^Nontaxane regimens included cisplatin- and epirubicin-based regimens.
